# Photon-counting CT-derived hepatic extracellular volume quantification for noninvasive risk stratification of clinically significant portal hypertension (CSPH): a prospective cohort study

**DOI:** 10.1007/s00330-025-12222-8

**Published:** 2025-12-23

**Authors:** Tatjana Dell, Verena Tischler, Dario Zocholl, Narine Mesropyan, Alice Margarida Jacob, Johannes Chang, Bernhard Schmidt, Claus Christian Pieper, Alexander Isaak, Patrick Kupczyk, Carsten Meyer, Julian Luetkens, Christian Strassburg, Christian Jansen, Daniel Kuetting

**Affiliations:** 1 Department of Diagnostic and Interventional Radiology and Quantitative Imaging Lab Bonn (QILaB), University Hospital Bonn, Bonn, Germany; 2 Institute of Pathology, University Hospital Bonn, Bonn, Germany; 3 Institute of Medical Biometry, Informatics and Epidemiology, Medical Faculty, University of Bonn, Bonn, Germany; 4 Department of Internal Medicine I, Center for Cirrhosis and Portal Hypertension Bonn (CCB), University Hospital Bonn, Bonn, Germany; 5 Computed Tomography, Siemens Healthcare GmbH, Forchheim, Germany

**Keywords:** Clinically significant portal hypertension (CSPH), Liver stiffness measurement (LSM), Photon-counting CT (PCCT), Transient elastography (TE), Hepatic extracellular volume (ECV)

## Abstract

**Objectives:**

To evaluate whether photon-counting CT (PCCT)-derived hepatic extracellular volume (ECV) can serve as a noninvasive imaging biomarker to detect or exclude clinically significant portal hypertension (CSPH) in patients with compensated advanced chronic liver disease (cACLD).

**Materials and methods:**

This prospective single-center study included 113 participants with chronic liver disease who underwent contrast-enhanced liver PCCT between February 2022 and January 2025. Hepatic ECV was calculated from the delayed phase (5 min post-contrast). Liver stiffness measurements (LSM) by transient elastography (*n* = 79) and histological fibrosis grading (*n* = 34) served as reference standards. Correlations were evaluated using Spearman’s ρ, and multivariable linear regression was applied to identify independent associations with LSM. Diagnostic performance for CSPH was assessed with ROC analysis using guideline-endorsed LSM thresholds (≤ 15 kPa to rule out; ≥ 25 kPa to rule in).

**Results:**

Hepatic PCCT-ECV showed strong correlations with fibrosis grade (ρ = 0.79, *p* < 0.001) and LSM (ρ = 0.83, *p* < 0.001). An ECV threshold of 27.7% identified CSPH (LSM ≥ 25 kPa) with 95% sensitivity and 93% specificity. To rule out CSPH (LSM ≤ 15 kPa), a threshold of 23.9% achieved 88% sensitivity and 97% specificity. In multivariable analysis including MELD score and platelet count, ECV remained independently associated with LSM. Inter-observer reproducibility was good (two-way random-effects, absolute agreement ICC = 0.83).

**Conclusion:**

PCCT-derived ECV provides a promising noninvasive biomarker for identifying or excluding CSPH in patients with chronic liver disease. Given its reproducibility and integration into routine HCC surveillance imaging, ECV may support early risk stratification. Validation in multicenter settings is warranted.

**Key Points:**

***Question*** *Can a quantitative biomarker from routine CT scans reliably stratify risk for clinically significant portal hypertension, overcoming limitations of current noninvasive methods?*

***Findings*** *Photon-counting CT-derived ECV accurately stratifies portal hypertension risk, offering high sensitivity (95%) for rule-in and high specificity (97%) for rule-out.*

***Clinical relevance*** *Integrating PCCT-ECV into routine surveillance CT provides an opportunistic, noninvasive tool for CSPH risk stratification, guiding timely patient management without requiring a separate examination.*

**Graphical Abstract:**

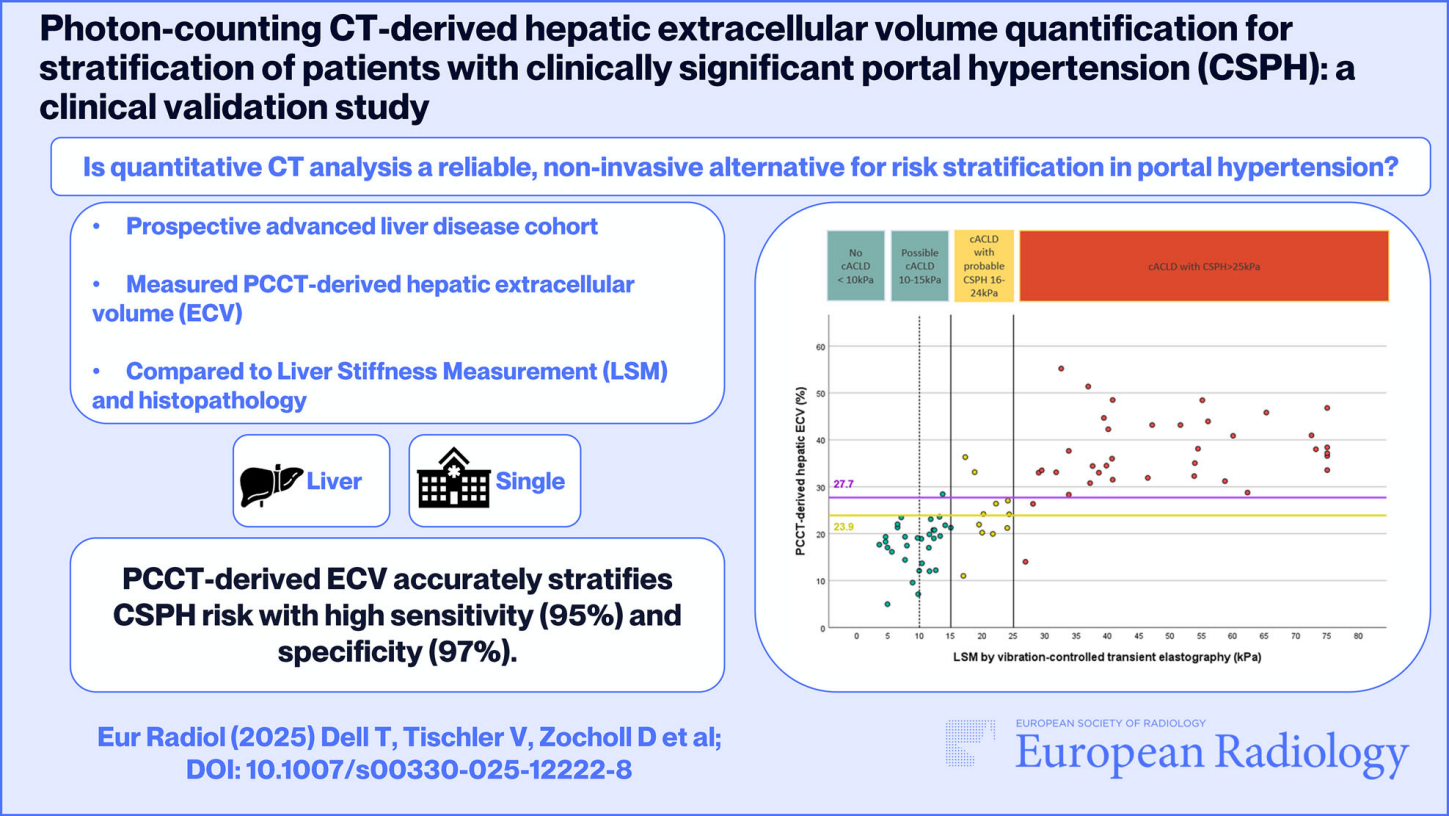

## Introduction

Chronic liver disease (CLD) and associated cirrhosis are among the leading causes of mortality worldwide, driven primarily by chronic HCV, chronic HBV, alcohol-associated liver disease and metabolic steatotic liver disease (MASLD), the latter two with increasing prevalence [1, 2]. Advanced CLD (ACLD) progresses from a compensated stage, often asymptomatic, to decompensated cirrhosis with poor survival [3]. While median survival in the compensated stage exceeds 12 years, 5–8% of patients decompensate each year, and a subset may succumb to their first decompensating event [4].

In patients with compensated advanced chronic liver disease (cACLD), the onset of clinically significant portal hypertension (CSPH) marks a critical transition, substantially increasing the risk of severe complications. These include ascites, portal hypertensive variceal bleeding, and hepatic encephalopathy [4, 5]. The reference standard for diagnosing CSPH is hepatic venous pressure gradient (HVPG) measurement, but its invasiveness and limited availability restrict its routine use [6]. Given these constraints, contemporary guidance (e.g., Baveno VII and recent AASLD practice statements) endorses noninvasive surrogates for CSPH risk, noninvasive liver disease assessment (NILDA) – chiefly liver stiffness measurement (LSM), which correlates with HVPG and provides pragmatic rule-in/rule-out thresholds (≥ 25 kPa; ≤ 15 kPa) [5, 7–9].

Despite the growing use of elastography, significant limitations remain. LSM is operator-dependent, requires dedicated equipment, and can be unreliable in patients with obesity or ascites [10]. Consequently, there is an unmet need for further widely accessible and objective imaging markers to improve CSPH risk stratification. In patients undergoing regular hepatocellular carcinoma (HCC) surveillance at defined intervals, the corresponding imaging studies represent an opportunity to extract additional clinically relevant information beyond tumor detection.

Recent studies assessing the value of CT in the evaluation of CSPH have primarily relied on morphological analysis [11, 12]. However, such qualitative approaches have inherent limitations in sensitivity and reproducibility. Imaging-based deduction of hepatic extracellular volume (ECV) has emerged as a promising surrogate marker, demonstrating a strong correlation with fibrosis severity in previous MRI-based studies [13, 14]. Spectral CT imaging enables precise ECV quantification—as recently demonstrated for photon-counting CT (PCCT) in myocardial imaging, acquired in a delayed equilibrium phase. Compared with conventional energy-integrating and standard dual-energy CT, PCCT provides intrinsically higher spectral separation via direct photon conversion. This reduces beam-hardening and cross-talk, improves iodine density quantification at routine dose levels, and thereby enables more precise and reproducible ECV estimation [15]. The value of CT-derived ECV for clinical grading of cirrhosis (including CSPH) is not yet determined.

This study aims to evaluate whether PCCT-derived ECV can stratify the risk of CSPH using noninvasive, guideline-supported surrogates (LSM thresholds) and histology to contextualize fibrosis severity, thereby complementing established NILDA pathways.

## Materials and methods

### Study participants

This prospective study was approved by an institutional review board (Ethics Committee of the Medical Faculty, University of Bonn, application number 254/22). Participants gave written informed consent.

**Fig. 1:**
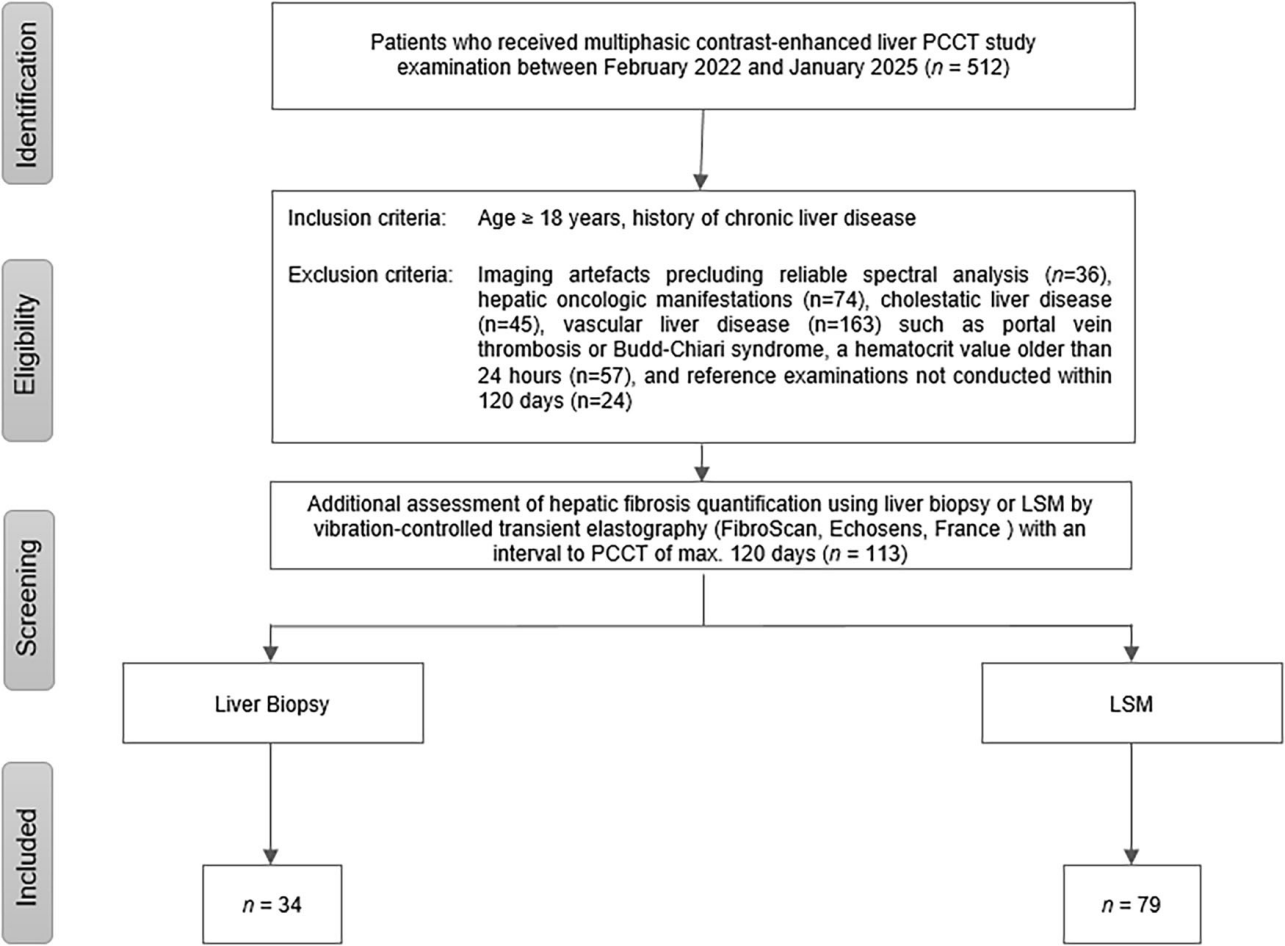
Flow diagram of participant inclusion and exclusion. LSM, liver stiffness measurement; PCCT, photon-counting CT

Study enrollment is depicted in Fig. 1. Patients undergoing multiphasic contrast-enhanced liver study PCCT examination between February 2022 and January 2025 were eligible for inclusion. Inclusion criteria comprised age ≥ 18 years and a history of chronic liver disease. Exclusion criteria included imaging artifacts precluding reliable spectral analysis, hepatic malignancies, and cholestatic or vascular liver disease. Participants eligible for inclusion underwent at least one of the following comparative modalities for hepatic fibrosis quantification within 120 days of PCCT imaging: liver biopsy or elastography. Hematocrit (Hct) for ECV calculation was derived from a peripheral venous sample obtained within 24 h of PCCT.

Demographics and medical history were retrieved from medical records.

### Liver PCCT imaging study protocol

All examinations were clinically indicated and acquired on a dual-source PCCT system (NAEOTOM Alpha, Siemens Healthineers). All examinations were acquired at 120 kVp in a triphasic liver protocol per institutional standard, with phases acquired at fixed delays: arterial phase (time from bolus tracking, 18 s), portal venous phase (time from bolus tracking, 50 s), and delayed parenchymal phase (time from bolus tracking, 300 s, equilibrium phase). Images were reconstructed with a slice thickness of 1 mm using a spectral soft tissue kernel (Qr40; QIR-4; Image Quality Level: 113 keV). All examinations were reconstructed in spectral processing file format to allow for spectral analysis. Only the 300-s delayed (equilibrium) phase was used for ECV computation.

### Quantitative ECV measurement

**Fig. 2:**
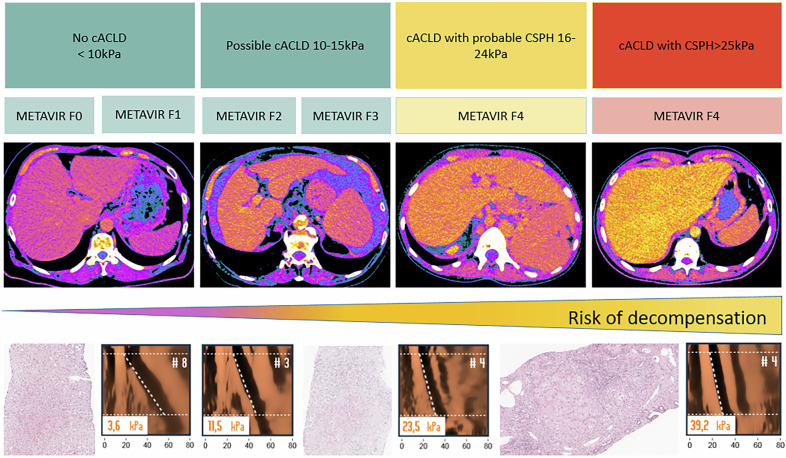
Hepatic ECV quantification in participants with varying degrees of hepatic fibrosis to cirrhosis. ECV values measured in regions of interest (circles) in the right liver lobe on axial equilibrium contrast-enhanced PCCT iodine maps (first row), shown with corresponding LSM by transient elastography (second row) and photomicrographs (hematoxylin-eosin stain; scale bar = 50 μm) of histologic samples (third row). cACLD, compensated advanced chronic liver disease; CSPH, clinically significant portal hypertension; ECV, extracellular volume; PCCT, photon-counting CT; LSM, liver stiffness measurement

Spectral-based image data from the equilibrium phase were post-processed using a dedicated workstation (syngo.via, version VB80D; Siemens Healthineers) to generate iodine density images (Fig. 2). Iodine density measurements were performed by an experienced radiologist (T.D.) with 12 years of expertise in abdominal imaging. Regions of interest (ROIs) with at least a 1 cm radius were placed in four predefined liver segments (III, IVa, V and VII), ensuring the exclusion of major vessels, bile ducts, liver borders, and artifacts. The mean iodine density of the liver parenchyma (I_liver) was calculated as the average of these four segmental measurements. The iodine density of the aorta (I_aorta) was measured at the level of the ostium of the truncus coeliacus. Based on these values, I-ratios and CT-ECV were calculated using the following formulas:

$${I}_{{ratio}}={I}_{{liver}}\div{I}_{{aorta}}$$

$${CT\; ECV}\left( \% \right)=\left(1-{hematocrit}\right)\times {I}_{{ratio}}\times 100$$

where I_liver is the mean iodine density (mg/mL) from four ROIs (segments III, IVa, V, VII) and I_aorta is the aortic iodine density measured at the celiac trunk level; Hct is the hematocrit (fraction) from peripheral venous blood obtained within ≤ 24 h of PCCT. This equilibrium, iodine-based approach obviates the need for a non-contrast scan and follows established equilibrium CT methodology and spectral iodine mapping reports.

To assess inter-observer agreement, a random subset of 20 examinations was re-measured by a second abdominal radiologist (D.K.), blinded to the first reader. Both readers independently placed ROIs per protocol on delayed iodine maps. Agreement was quantified using two-way random-effects ICC (absolute agreement, single-measure).

### Liver biopsy and histologic analysis

Clinically indicated right-lobe liver core needle biopsies were performed from a transjugular access with an 18-gauge needle; at least 3 biopsies were taken per participant. A hepatopathologist (V.T., with 7 years of experience in gastrointestinal pathology) evaluated the samples while being blinded to clinical and radiological data. Each biopsy specimen was meticulously embedded in paraffin wax and fixed in formalin solution, ensuring optimal preservation for subsequent histological examination. An inadequate biopsy sample was defined as having a core length less than 1.5 cm. Liver fibrosis was staged according to the METAVIR grading system as follows: F0: No fibrosis, F1: Portal fibrosis without septa, F2: Portal fibrosis with few septa, F3: Numerous septa and incomplete nodules, and F4: Complete nodules (liver cirrhosis) [16].

### Liver stiffness measurement

LSM were acquired using vibration-controlled transient elastography (FibroScan, Echosens) and reported as the median value in kPa. LSMs were performed by an experienced gastroenterologist (C.J., with 12 years of experience in cACLD) according to the manufacturer’s instructions. Measurements were considered valid if 10 consecutive readings yielded an interquartile range (IQR)/median ratio of < 30%. Using a standard 3.5 MHz probe (≥ 2.5 cm target-to-skin distance, XL probe; < 2.5 cm target-to-skin distance, M probe), the right liver lobe was assessed from an intercostal position, the left lobe in the epigastric region with the participant in the supine position. According to the Baveno VII criteria, participants were classified into two groups: assumed CSPH (LSM ≥ 25 kPa) or excluded CSPH (LSM ≤ 15 kPa). In line with Baveno VII, these thresholds were used as noninvasive surrogates for CSPH in all diagnostic accuracy analyses.

### Statistical analysis

Statistical analysis was performed using SPSS, version 26 (IBM), and R, version 4.3.3.

Participant demographic and imaging information were summarized using descriptive statistics. Categorical variables are expressed as numbers and percentages and continuous variables as means ± standard deviations.

The Spearman ρ coefficient was used to assess the correlation between PCCT- ECV measurements and those from biopsy and LSM.

ROC analyses were performed to assess discrimination of PCCT-derived hepatic ECV for CSPH (outcome defined by guideline-endorsed LSM strata: ≤ 15 vs ≥ 25 kPa). AUCs with 95% confidence intervals were estimated based on 10,000 bootstrap samples. For internal validation, 5-fold cross-validation was performed and repeated 1000 times to estimate cross-validated AUC (mean ± SD, 95% CI), and Harrell’s optimism-corrected AUC was calculated based on 1000 bootstrapped samples. In the biopsy subset (outcome: METAVIR F3–F4 vs ≤ F2), pairwise AUC comparisons between ECV and LSM were conducted using the DeLong test.

Decision-curve analysis (DCA) quantified clinical net benefit over threshold probabilities 0.10–0.60, comparing an ECV-based model against “treat-all” and “treat-none” strategies (and, in sensitivity analysis, an LSM-based reference curve). Covariates were pre-specified to balance biological plausibility and model parsimony. Although age and sex were evaluated, they showed no meaningful association with ECV or LSM in exploratory checks and were not retained to avoid overfitting; adjustment focused on platelet count and MELD, which directly relate to portal hypertension pathophysiology.

## Results

Among the initial 512 patients considered for inclusion, 339 patients were excluded for various reasons: imaging artifacts (*n* = 36), hepatic oncologic manifestations (*n* = 74), cholestatic liver disease (*n* = 45), vascular liver disease (*n* = 163) such as portal vein thrombosis or Budd-Chiari syndrome, a hematocrit value older than 24 h (*n* = 57), and reference examinations not conducted within 120 days (*n* = 24). The final analysis included 113 participants (Fig. 1). Demographic and clinical characteristics of the participants are shown in Table 1. Most patients in the cohort had alcohol-associated chronic liver disease, accounting for 46% (52 out of 113).

**Table 1:** Characteristics of study participants (*n* = 113) stratified by comparison examination

	Biopsy *n* = 34		LSM *n* = 79
Sex^a^			
M	25 (67)		49 (62)
F	9 (26)		30 (38)
Mean age^b^			
	54.7 ± 13.2		58.8 ± 13.9
Interval to PCCT (d)^b^			
	15 ± 15.2		17 ± 24.6
Fibrosis grade (METAVIR)^a^		Liver stiffness	
F0	2 (6)	≤ 15 kPa	30 (38)
F1	3 (9)	16–24 kPa	12 (15)
F2	2 (6)	≥ 25 kPa	37 (47)
F3	9 (26)		
F4	18 (53)		
Genesis of CLD			
Alcohol-associated	15 (44)		36 (46)
Chronic viral hepatitis	1 (3)		4 (5)
Metabolic steatotic liver disease	5 (15)		6 (8)
Autoimmune hepatitis	0		2 (2)
Genetic disorders	2 (6)		11 (14)
Drug-induced liver injury	3 (9)		1 (1)
Cryptogenic	8 (23)		19 (36)

^a^ Data are numbers of patients, with percentages in parentheses.

^b^ Data are mean ± SD

*LSM* liver stiffness measurement, *PCCT* photon-counting CT, *CLD* chronic liver disease

### Liver stiffness measurement

Of the 113 included participants, 79 (mean age, 58.8 years ± 13.9 [SD]; age range, 20–85 years; 30 [38%] female participants, 49 [62%] male participants) underwent LSM and PCCT, with a mean interval between examinations of 17 days ± 24.6 (range, 0–95 days). Of these participants, 47% (37 of 79) had an LSM ≥ 25 kPa, 38% (30 of 79) had an LSM ≤ 15 kPa, and 15% (12 of 79) had an LSM > 16 kPa and < 25 kPa. The mean LSM was 29 kPa (± 22 [SD]; range, 4–75 kPa).

### Biopsy

Of the 113 included participants, 34 (mean age, 54.7 years ± 13.2; age range, 23–72 years; nine [26%] female participants, 25 [74%] male participants) underwent liver biopsy. The interval between biopsy and PCCT ranged from 0 to 63 days (mean, 15 days ± 15.2). As summarized in the table, 53% (18 of 34) of the included participants had a fibrosis grade of 4, 26% (9 of 34) had F3, 6% (2 of 34) had F2, 9% (3 of 34) had F1, and 6% (2 of 34) had no fibrotic transformation.

### Correlation analyses

**Fig. 3:**
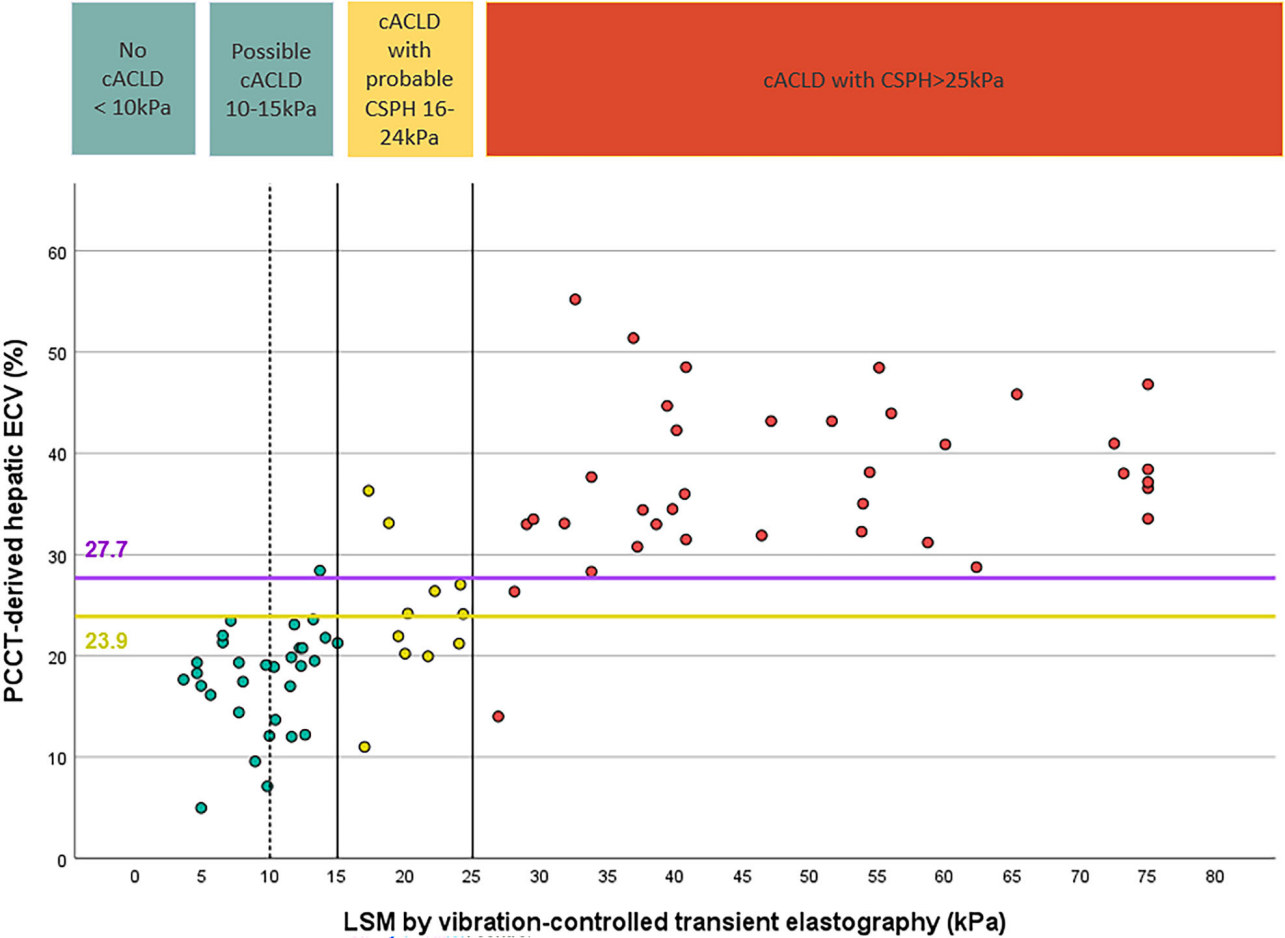
Scatter plot showing PCCT-derived hepatic ECV (%) versus liver stiffness (kPa) by transient elastography. The dashed line at 10 kPa separates no cACLD from possible cACLD. Solid lines at 15 and 25 kPa mark thresholds for ruling in/out CSPH. Colors indicate cACLD stages. Two horizontal lines indicate PCCT-ECV thresholds: the yellow line at 23.9% represents the rule-out threshold for CSPH (in patients with LSM ≤ 15 kPa), while the purple line at 27.7% marks the rule-in threshold for CSPH. PCCT, photon-counting CT; ECV, extracellular volume; cACLD, compensated advanced chronic liver disease; CSPH, clinically significant portal hypertension; LSM, liver stiffness measurement

**Fig. 4:**
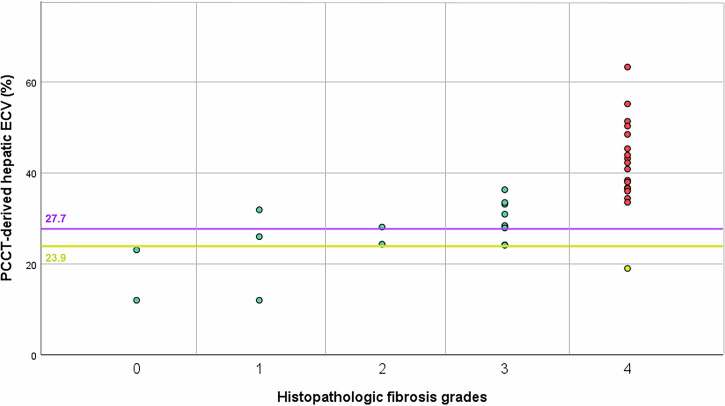
Scatter plot showing PCCT-derived hepatic ECV (%) across histopathologic fibrosis grades (METAVIR 0–4). Two horizontal lines indicate PCCT-ECV thresholds: the yellow line at 23.9% represents the rule-out threshold for CSPH (in patients with LSM ≤ 15 kPa), while the purple line at 27.7% marks the rule-in threshold for CSPH. Data points are color-coded by fibrosis severity. PCCT, photon-counting CT; ECV, extracellular volume; CSPH, clinically significant portal hypertension

The Spearman ρ correlation coefficient was 0.83 between LSM and PCCT-evaluated ECV (*p* < 0.001) and 0.79 between histologic grades and PCCT-ECV (*p* < 0.001) (Figs. 3, 4). In exploratory analyses, age and sex did not materially alter the ECV–LSM association or discrimination (data not shown). In a predefined subset assessed by an independent second reader, hepatic ECV measurements showed good agreement with the primary reader (two-way random-effects, absolute-agreement, single-measure ICC = 0.83; 95% CI [0.68–0.91]).

### Receiver operating characteristic analysis

**Fig. 5:**
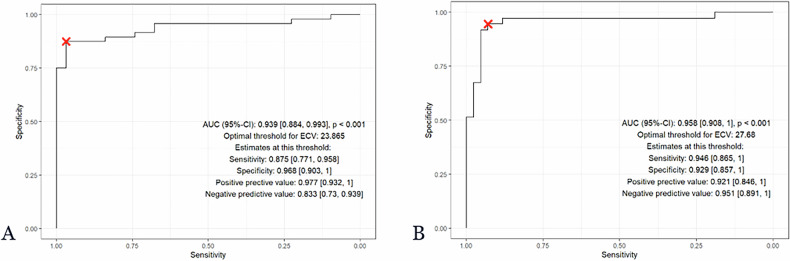
ROC curve: ECV for CSPH (LSM ≤ 15 (**A**) vs ≥ 25 kPa (**B**)). ECV, extracellular volume; CSPH, clinically significant portal hypertension; LSM, liver stiffness measurement

ROC curves for CSPH classification (LSM ≤ 15 vs ≥ 25 kPa) using ECV are shown in Fig. 5, with corresponding AUCs (95% CIs) in Table 2.

**Table 2:** Cross-validated and optimism-corrected AUCs (95% confidence intervals) of the ECV model for CSPH at LSM cutoffs of ≤ 15 kPa and ≥ 25 kPa

LSM cutoff	Cross-validated AUC (± SD)	95% CI (empirical across folds)	Apparent AUC	Optimism-corrected AUC
≤ 15 kPa	0.939 ± 0.060	0.800–1.000	0.939	0.938
≥ 25 kPa	0.958 ± 0.054	0.818–1.000	0.958	0.957

*ECV* extracellular volume, *CSPH* clinically significant portal hypertension, *LSM* liver stiffness measurement

The threshold for PCCT-ECV that best identifies patients with CSPH was 27.7%. With this threshold, 35 of 37 patients with LSM-based diagnosis of CSPH as well as 39 of 42 patients with ruled out CSPH were correctly identified, corresponding to sensitivity of 94.6% (95% CI [0.865, 1]) and specificity of 92.9% (0.857, 1), as well as positive predictive value of 92.1% (0.846, 1) and negative predictive value of 0.951 (0.891, 1). Cross-validated AUC was 0.958 ± 0.054 (0.818, 1), optimism-corrected AUC was 0.958 (Table 2). A threshold of 23.9% was identified to rule out CSPH in patients with LSM ≤ 15 kPa. Applying this cutoff, a PCCT-ECV value below 23.9% yielded a sensitivity of 88% (95% CI [0.752, 0.958]), a specificity of 97% (95% CI [0.890, 0.998]), a positive predictive value of 0.977 (95% CI [0.932, 1]), and a negative predictive value of 0.833 (95% CI [0.725, 0.939]). Cross-validated AUC was 0.939 ± 0.06 (0.800; 1), optimism-corrected AUC was 0.938 (Table 2). Both threshold values are included in Fig. 4, which plots liver ECV measured with PCCT and LSM for each study participant, stratified by LSM according to Baveno criteria. ROC curves for CSPH classification (LSM ≤ 15 vs ≥ 25 kPa) using ECV are shown in Fig. 5.

**Fig. 6:**
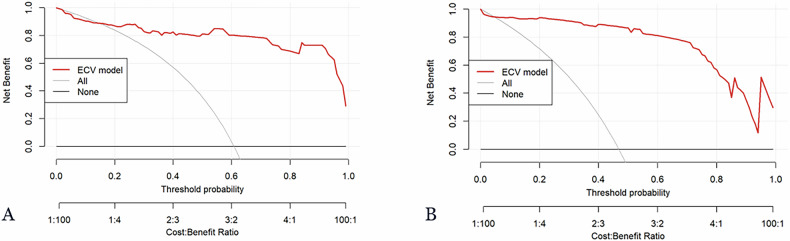
Decision-curve analysis of the ECV model vs treat-all/none strategies for CSPH. **A** LSM ≤ 15 kPa (rule-out). **B** LSM ≥ 25 kPa (rule-in). ECV, extracellular volume; CSPH, clinically significant portal hypertension; LSM, liver stiffness measurement

DCA indicated a positive net benefit of ECV across threshold probabilities 0.10–0.60 (Fig. 6).

## Discussion

This study evaluated the clinical value of hepatic PCCT-ECV quantification as a noninvasive tool for assessing hepatic fibrosis, comparing its performance to established NILDA methods, specifically elastography-based liver stiffness measurements using TE, as well as histological findings. Focusing on patients with CLD, PCCT-ECV showed a strong correlation with TE-based liver stiffness measurements (ρ = 0.83) and histologic fibrosis grades (ρ = 0.79).

Histopathology remains the reference standard for the diagnosis and staging of liver fibrosis, typically using systems such as the METAVIR score [17]. Our results are in good agreement with previous research showing a similarly strong correlation between CT-derived ECV (based on Hounsfield units) and quantitative histological analysis using digital image analysis and collagen quantification [18]. This strong correlation is preserved when ECV is derived from iodine density maps, as performed in the present study. Yoon et al demonstrated that iodine-based ECV exhibits an even stronger correlation with histopathologic grading compared to HU-based ECV [19]. In their study, an equilibrium phase was acquired 3 min after contrast administration, and an ECV-iodine cutoff of 29% was identified for the detection of cirrhosis—higher than the 27.7% cutoff determined in our cohort for the identification of CSPH, where a 5-min delay was employed for the equilibrium phase. These findings underscore that ECV values may be influenced by the timing of the equilibrium phase, a factor that remains insufficiently explored for hepatic imaging and warrants further investigation [20].

Although several studies have investigated CT-derived ECV, based either on Hounsfield units or iodine quantification, for the assessment of clinically significant fibrosis or cirrhosis, ECV-based risk stratification specifically in relation to CSPH in a CLD population has not yet been evaluated.

This gap is particularly relevant given that histopathology, although considered the gold standard for fibrosis staging, has limited utility in differentiating advanced stages of liver disease. In patients with cACLD—especially those at risk of decompensation or with established CSPH—histopathology cannot reliably distinguish between clinical stages [21, 22]. Historically, cirrhosis has been regarded as an “end-stage” condition; however, it is now recognized that cirrhosis represents a dynamic disease spectrum ranging from compensated to severely decompensated stages (22). This clinical variability is also reflected in the heterogeneity of ECV values observed within histological F4 stages, both in our study (Fig. 3) and in previous studies such as that of Yoon et al [19].

Given its ability to quantitatively characterize the continuum of fibrotic remodeling, CT-derived ECV may therefore serve as a more nuanced biomarker, enabling more precise stratification of patients across the spectrum of cirrhosis beyond what is achievable with conventional histopathologic grading. Early identification of CSPH is crucial, as timely initiation of treatments such as carvedilol or non-selective beta-blockers can significantly improve patient outcomes [23]. On the other hand, reliably ruling out CSPH could also be of great value for optimizing medical resource allocation and avoiding unnecessary invasive interventions.

Traditionally, CSPH was diagnosed using hepatic venous pressure gradient (HVPG) measurement, a minimally invasive but highly prognostic tool in cACLD, capable of stratifying the risk of hepatic decompensation and liver-related mortality [24]. HVPG values ≤ 5 mmHg are considered normal; 6–9 mmHg indicates subclinical portal hypertension, and values ≥ 10 mmHg define CSPH [8]. However, despite its clinical value, HVPG measurement is invasive, technically demanding, and not widely available, which has led to the growing adoption of NILDA tools to evaluate portal hypertension and guide patient management. Several noninvasive markers—including blood-based tests and LSM—show prognostic performance comparable to HVPG. Current guidelines, including Baveno VII and AASLD 2024, endorse LSM-based stratification [1, 25–27]. Specifically, LSM < 10 kPa rules out cACLD, while ≥ 15 kPa confirms the diagnosis. The “Rule of Five” proposes pragmatic thresholds for CSPH: (1) LSM > 25 kPa, (2) LSM 20–24 kPa with platelets < 150 × 10⁹/L, (3) LSM 16–20 kPa with platelets < 110 × 10⁹/L. CSPH can be excluded with LSM ≤ 15 kPa and platelets ≥ 150 × 10⁹/L. Even when platelet count is not included in the risk stratification of patients with cACLD, the LSM thresholds of 15 kPa and 25 kPa remain highly specific for confirming or excluding CSPH [28]. Our findings support this concept by demonstrating that PCCT-ECV alone provides robust diagnostic performance. A threshold of 27.7% yielded 94.6% sensitivity and 92.9% specificity for ruling in CSPH, while a threshold of 23.9% applied in patients with LSM ≤ 15 kPa allowed CSPH to be ruled out with 88% sensitivity and 97% specificity.

Unlike MRI-derived ECV, PCCT-ECV is obtained from a single delayed equilibrium acquisition without pre-/post-contrast T1 mapping, resulting in shorter scanner time and streamlined post-processing. In our practice, this delayed equilibrium phase is already part of the standard multiphasic liver CT protocol for HCC surveillance; thus, hepatic ECV is derived opportunistically from routine imaging without additional appointments or protocol modifications, reducing patient burden and improving workflow throughput.

Thus, PCCT-ECV can serve as an additional noninvasive tool for assessing CSPH in various clinical contexts. It can be employed either in the surveillance for HCC or in the imaging of HCC itself. In patients with HCC undergoing tumor resection, portal hypertension has been identified as a highly predictive factor for the risk of postoperative liver decompensation [29, 30]. Consequently, current EASL guidelines recommend considering portal hypertension in treatment decision-making for patients with resectable HCC [31]. Moreover, CSPH has been recognized as an important prognostic factor in patients with HCC undergoing transarterial chemoembolization [32, 33]. Previous studies evaluating CT-based appraisal of CSPH have primarily focused on structural assessments and morphological features such as splenomegaly, gastroesophageal varices, spontaneous portosystemic shunts, or ascites [11, 12]. In contrast, CT-based ECV constitutes a quantitative, reproducible surrogate marker, offering a more objective and precise approach for evaluating CSPH.

This study has several limitations that must be acknowledged. First, the reference standard methods employed—transient elastography (TE)-based liver stiffness measurement and liver biopsy—have limitations in diagnostic accuracy [34, 35]. Liver biopsy, while often regarded as a gold standard, provides only a semiquantitative assessment and is limited by sampling error, especially as tissue was obtained from a single liver segment, which may not adequately reflect the heterogeneous distribution of fibrosis. Similarly, TE accuracy is influenced by both operator- and patient-dependent factors, including obesity, hepatic inflammation, and narrow intercostal spaces. In TE, measurements are typically taken from the area of maximal stiffness, which may introduce bias and limit reproducibility. In contrast, PCCT-ECV measurement lacks real-time feedback during region-of-interest (ROI) placement. The observed good inter-observer agreement for ECV (ICC = 0.83) supports the reproducibility of the standardized ROI protocol; nevertheless, larger multi-reader, multicenter evaluations remain warranted to confirm generalizability.

Another limitation is the limited availability of PCCT technology, which is currently accessible only at select academic and high-end imaging centers. Thus, the potential clinical utility of PCCT-based liver assessment remains confined to these specialized settings. Additionally, this study was conducted at a single institution using a single scanner, which may affect generalizability. Larger, multicenter studies with independent cohorts are needed to validate the findings and confirm the clinical value of contrast-enhanced liver PCCT in broader populations. We used fixed post-trigger delays for the delayed phase (300 s). While individualized phase timing (e.g., repeated bolus triggering) might further optimize parenchymal enhancement in cACLD, it increases operational complexity, scan time, and variability across patients and centers. Future studies should compare fixed versus hemodynamically adapted timing schemes.

The chosen time window of up to 120 days between PCCT acquisition and reference standard assessment presents another limitation. Although cirrhotic disease progression is typically slow, major lifestyle modifications or therapeutic interventions within this interval could have influenced hepatic fibrosis status. Given the sparse literature on optimal timing for such correlations, this cutoff represents a pragmatic but imperfect compromise between data availability and pathophysiological stability. Another limitation of this study is the composition of the study cohort, in which 46% of patients had alcohol-associated liver disease, while other etiologies of cirrhosis were represented only in small numbers. In addition, the proportion of patients with cryptogenic cirrhosis was notably high. Accordingly, etiology-specific ECV distributions and thresholds could not be reliably estimated in this cohort. As disease-specific pathophysiological differences may influence ECV values, future studies will be necessary to validate PCCT-ECV quantification separately for different underlying causes of cirrhosis. While age and sex were not retained in adjusted analyses due to lack of association and to preserve parsimony, residual confounding cannot be excluded and merits assessment in larger, multicenter cohorts. Finally, PCCT-ECV quantification was not specifically assessed for risk stratification within the “gray zone” between confidently excluded and confirmed CSPH. Notably, LSM certainty attenuates in the 16–24 kPa range, where recommendations are conditional (e.g., platelet count and clinical context) and the evidentiary base is less stable than at rule-out/rule-in thresholds; to minimize spectrum bias and circularity, we therefore anchored ROC analyses to high-certainty strata (≤ 15 and ≥ 25 kPa). Consistent with guideline practice, we adopted a dual-threshold strategy to provide clinically actionable rule-out/rule-in decisions rather than impose a single compromise cutoff that could increase misclassification. Consequently, patients with intermediate LSM (16–24 kPa)—and analogously those with intermediate ECV values—require integrated NILDA assessment, with PCCT-ECV interpreted as a supportive adjunct rather than a stand-alone determinant. Future studies should derive and externally validate continuous ECV-based risk models and evaluate reclassification performance and decision-curve net benefit specifically within the gray zone.

In conclusion, our study demonstrated that PCCT-based imaging not only provides established morphological information but also enables quantitative assessment of cirrhosis through ECV measurement and facilitates the detection or exclusion of CSPH in a routine clinical setting. These findings suggest potential applications in disease screening, monitoring, and prevention. Further prospective multicenter studies are essential to validate PCCT-ECV quantification as a biomarker for fibrosis and cirrhosis, and to establish optimal thresholds for clinical use and integration into clinical trials.

## Data Availability

Data generated or analyzed during the study are available from the corresponding author upon reasonable request.

## Abbreviations

ACLD: Advanced chronic liver disease
cACLD: Compensated advanced chronic liver disease
CLD: Chronic liver disease
CSPH: Clinically significant portal hypertension
ECV: Extracellular volume
HVPG: Hepatic venous pressure gradient
LSM: Liver stiffness measurement
NILDA: Noninvasive liver disease assessment
PCCT: Photon-counting CT
ROC: Receiver operating characteristic
TE: Transient elastography
